# Subsurface Banding of Poultry Manure Enhances Photosynthetic Efficiency, Yield, and Nutrient Uptake in Buckwheat

**DOI:** 10.3390/plants14172700

**Published:** 2025-08-29

**Authors:** Sina Fallah, Hossein Abedini Dastgerdi, Hans-Peter Kaul, Aliyeh Salehi

**Affiliations:** 1 Department of Agronomy, Faculty of Agriculture, Shahrekord University, Shahrekord 115, Iran; falah1357@yahoo.com (S.F.);; 2 Institute of Agronomy, Department of Agricultural Sciences, BOKU University, Konrad Lorenz-Straße 24, 3430 Tulln an der Donau, Austria; 3 Institute of Organic Farming, Department of Agricultural Sciences, BOKU University, Gregor-Mendel Straße 33, 1180 Vienna, Austria

**Keywords:** nutrient uptake, organic fertilization, photosynthetic pigments, poultry manure, subsurface banding, sustainable agriculture

## Abstract

Manure application may improve plant growth, yield, and ecological sustainability. This study investigates optimized organic fertilizer application methods for enhancing buckwheat (*Fagopyrum esculentum*) productivity in semi-arid conditions. Treatments include broadcasting (Br) and subsurface banding (Ba) of poultry (PM) and cattle (CM) manure and foliar spraying (S) of manure extracts (1:5 and 1:10 ratios), urea fertilizer (UF), and a control. Subsurface-banded poultry manure (BaPM) maximized chlorophyll *b* (4.0 µg/mL), carotenoids (2.30 µmol/mL), anthocyanin (0.02 µmol/mL), leaf area index (2.03), seed nitrogen (3.4%), and spikes per plant (17). BaPM achieved the highest seed yield (646 kg/ha), comparable to BrPM, BaCM, and SPM(1:5). The maximum seed phosphorus content (0.43%) was observed in the BaPM, BrPM, and SCM(1:10) treatments. Dry matter peaked under UF (4870 kg/ha) and BaPM (4641 kg/ha). Banding placement improved nutrient uptake by enhancing root zone retention, while foliar poultry extract (1:5) mitigated phosphorus deficiency. These findings demonstrate that integrating certain manure types with targeted application methods—particularly subsurface banding of poultry manure—optimizes nutrient use efficiency, crop performance, and environmental sustainability in buckwheat cultivation.

## 1. Introduction

Sustainable production of healthy food while considering social, economic, and environmental factors has become a significant topic in agriculture, ecology, and environmental science [1]. In this regard, cropping systems should be redesigned to have a neutral or even positive environmental impact while contributing to healthy nutrition and food safety [2]. Various sustainable agricultural practices such as organic farming are being explored to achieve these goals [2]. This is partly due to escalating environmental issues linked with the application of synthetic fertilizers, such as their energy usage and production expenses, their negative impact on biological cycles, and the durability of agricultural ecosystems. These challenges necessitate a reassessment of strategies to enhance agricultural productivity [3].

Organic fertilizers are widely acknowledged for their environmental benefits in contrast to synthetic fertilizers, including improving soil organic matter, enhancing microbial activity, and promoting biodiversity [4,5]. Additionally, organic fertilizers play a vital role in supporting sustainable agriculture by enhancing crop production [6,7]. This makes them a key alternative to mineral fertilizers in organic systems [8].

Sustainable nutrient management, including application methods and rates, plays a critical role in enhancing the production and quality of crops. The conventional method of manure application, surface broadcasting by splash plate applicator, is rapid and inexpensive. Nonetheless, this distribution of manure frequently leads to inconsistent coverage and might not be easily available to the roots of plants [9]. Subsurface band application of solid manures has emerged as a viable alternative to traditional surface broadcasting [10]. This novel method successfully tackles numerous limitations linked to surface applications, such as loss of ammonium-N (NH_4_–N) fraction through ammonia volatilization, by positioning a measured quantity of manure in thin strips beneath the soil surface, usually adjacent to the planting row [11]. Tewolde et al. [12] found that subsurface banding of poultry manure conserves poultry manure-derived nitrogen and, compared to conventional surface broadcasting, facilitates its uptake by corn. This method also significantly reduces nutrient losses in surface runoff and leachate. For instance, subsurface banding has been shown to decrease phosphorus and nitrogen concentrations in runoff water by up to 96% [13] and to reduce ammonium-N, dissolved reactive phosphorus, and total suspended solids loadings by 67%, 73%, and 53%, respectively [14]. Amin et al. [15] reported that subsurface application of animal manure resulted in higher garlic yields compared to surface spreading. Foliar fertilization is a widely used supplementary strategy that delivers nutrients directly to the aerial parts of plants, allowing for rapid nutrient uptake and correction of deficiencies. Compared to soil fertilization, foliar application can improve nutrient use efficiency and reduce negative environmental impacts by targeting specific growth stages and nutrient needs [16]. Extensive evidence demonstrates that foliar fertilizers actively improve crop quality, yield, and metabolic processes [17].

Buckwheat (*Fagopyrum esculentum*), a gluten-free crop from the Polygonaceae family, is rich in phytochemicals that offer numerous health benefits [18]. It is grown in various ecological zones worldwide and is gaining popularity as a low-calorie, nutrient-dense food [19]. The bioactive compounds in buckwheat include flavonoids (such as rutin, quercetin, orientin, isoorientin, vitexin, and isovitexin), fatty acids, polysaccharides, proteins, amino acids, iminosugars, dietary fiber, fagopyrins, resistant starch, vitamins, and minerals [7,20]. Additionally, chlorophyll and anthocyanins are important traits in buckwheat that contribute to its nutritional value and health benefits. Chlorophyll plays a crucial role in photosynthesis, influencing plant growth and yield potential [21]. Anthocyanins, a class of flavonoids responsible for red to blue colors in plants, have gained attention for their potential health benefits. These compounds contribute to buckwheat’s high nutritional value and are known for their potential in preventing and treating various human diseases [19]. For these reasons, optimizing buckwheat cultivation through sustainable practices is essential. Furthermore, due to its high growth rate and short growing period, ensuring the provision of adequate nutrients is crucial for maximizing yield and quality. Proper utilization of animal manures has been shown to improve seed quality and minimize nutrient loss [7]. Specifically, Salehi et al. [22] demonstrated that the application of integrated fertilizers (3.8 Mg/ha) and broiler litter (7.5 Mg/ha) significantly enhances the productivity, nitrogen (N) and phosphorus (P) content, and nutrient use efficiency of buckwheat in semi-arid conditions. This highlights the importance of integrating organic amendments into farming practices for better crop performance while potentially enhancing chlorophyll and anthocyanin levels.

Overall, studies indicate that subsurface or banded application methods enhance nutrient utilization while minimizing environmental losses compared to traditional surface broadcasting techniques. However, to our knowledge, limited research exists comparing the effects of various application techniques on buckwheat production and nutritional value— such as broadcasting, subsurface banding, and foliar spraying—using both cattle manure and poultry manure with a synthetic fertilizer like urea. This gap is particularly relevant in semi-arid regions like Iran, where spring rains end early. Utilizing crops with shorter growth periods can provide significant benefits for farmers’ incomes and water conservation efforts while reducing groundwater extraction. Buckwheat, with its relatively rapid growth rate, presents a suitable option if the necessary nutrients for its growth are adequately supplied.

In this context, we hypothesize that the application method and nutrient source will significantly influence buckwheat performance. Specifically, we predict that subsurface banding and foliar application of cattle and poultry manures will outperform broadcasting and synthetic urea in enhancing buckwheat’s photosynthetic pigments, growth, yield, and macronutrient uptake by improving nutrient retention and availability in semi-arid calcareous soils. Our study aims to evaluate the application of animal manure in supporting optimal buckwheat production compared to synthetic fertilizers. Given the extensive applications and health benefits of buckwheat, we will investigate the effects of different animal manure application methods on photosynthetic pigments, growth parameters, yield, yield components, and macronutrient uptake in buckwheat.

## 2. Results

### 2.1. Plant Growth Parameters

**Figure 1:**
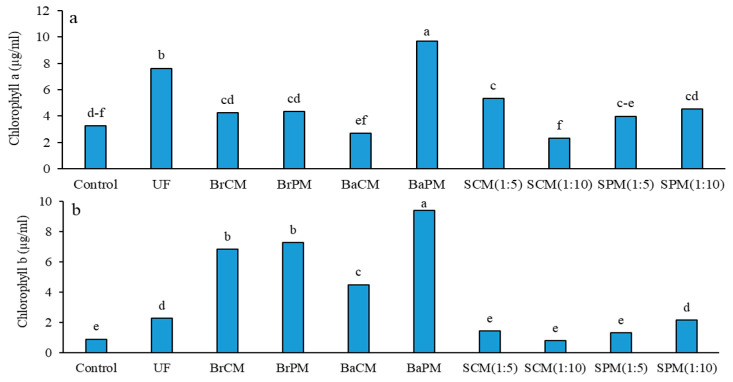
Effects of different fertilizer application methods on the concentrations of chlorophyll *a* (**a**) and chlorophyll *b* (**b**) in buckwheat. Control: without fertilizer; UF: urea fertilizer; BrCM: broadcasting of cattle manure; BrPM: broadcasting of poultry manure; BaCM: subsurface banding of cattle manure; BaPM: subsurface banding of poultry manure; SCM: foliar spraying of cattle manure at two levels (1:5 and 1:10); SPM: foliar spraying of poultry manure at two levels (1:5 and 1:10). Means followed by the same letter are not significantly different according to LSD test (*p* < 0.05).

Analysis of variance (ANOVA) indicated that fertilizer treatments had a significant effect on chlorophyll content (*p* < 0.01; Table S1). The highest chlorophyll *a* and chlorophyll *b* concentrations were noted in the BaPM treatment, whereas the lowest were found in the SCM(1:10) treatment (Figure 1a). The findings highlight the effectiveness of poultry manure versus cattle manure (55% increase), subsurface banding over broadcasting (44% increase), mineral fertilizers over organic fertilizers (39% increase), and solid application (subsurface banding and broadcasting) over foliar spraying (SCM and SPM; 30% increase) (Table S1; Figure 1a).

Across all application methods, poultry manure consistently produced higher chlorophyll *b* levels. Fertilization resulted in a threefold increase in leaf chlorophyll *b* levels compared to the control (4.0 vs. 0.92 µg/mL). Organic fertilizers accounted for 85% higher chlorophyll *b* levels than urea fertilizer. Subsurface banding and broadcasting of fertilizers displayed more than a threefold increase compared to foliar spraying treatments. Additionally, poultry manure treatments showed a 48% increase in chlorophyll *b* over cattle manure (Table S1; Figure 1b).

**Figure 2:**
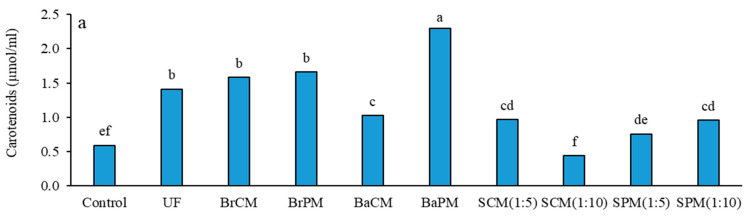

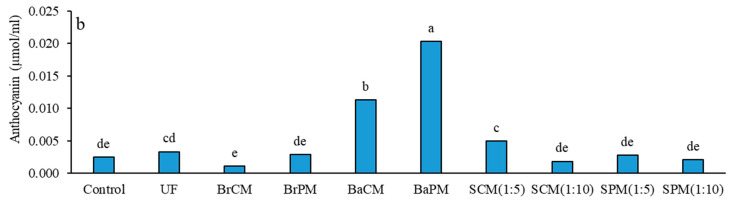
Effects of different manure application methods on the concentrations of carotenoids (**a**) and anthocyanin (**b**) in buckwheat. Control: without fertilizer; UF: urea fertilizer; BrCM: broadcasting of cattle manure; BrPM: broadcasting of poultry manure; BaCM: subsurface banding of cattle manure; BaPM: subsurface banding of poultry manure; SCM: foliar spraying of cattle manure at two levels (1:5 and 1:10); SPM: foliar spraying of poultry manure at two levels (1:5 and 1:10). Means followed by the same letter are not significantly different according to LSD test (*p* < 0.05).

The application of fertilizer treatments significantly affected the carotenoid and anthocyanin contents of buckwheat plants (*p* < 0.01; Table S1). The highest carotenoid concentration was found with BaPM treatment (2.30 µmol/mL), while the lowest was noted in the SCM(1:10) group (Figure 2a). Organic fertilizers caused 14% greater carotenoid levels than mineral fertilizers, although this difference was not statistically significant. Poultry manure treatments produced 41% higher carotenoid levels compared to cattle manure treatments. Furthermore, subsurface banding and broadcasting applications achieved 110% higher carotenoid concentrations than foliar spraying treatments (SCM, SPM). BaPM surpassed BaCM by 120% in carotenoids (Table S1; Figure 2a). The highest anthocyanin concentration was identified with BaPM (0.02 µmol/mL) and the lowest was recorded with BrCM (0.001 µmol/mL) (Figure 2b), while the assessment of anthocyanin content revealed significant differences (*p* < 0.01) across all orthogonal comparisons. Organic fertilizers increased anthocyanin levels by 78% compared to urea fertilizer, with poultry manure treatments surpassing cattle manure treatments by 46%. Subsurface banding achieved more than a sixfold increase in anthocyanin content compared to broadcasting, while subsurface banding and broadcasting fertilizers exceeded the efficacy of foliar spraying treatments by more than two times (Table S1; Figure 2b).

**Figure 3:**
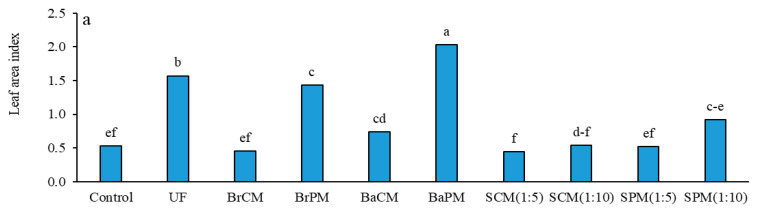

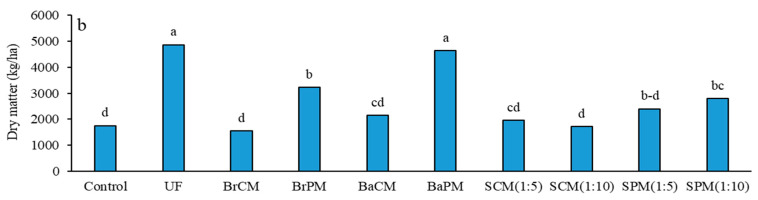
Effects of different fertilizer application methods on the leaf area index (LAI) (**a**) and dry matter (**b**) of buckwheat, measured 45 days after planting. Control: without fertilizer; UF: urea fertilizer; BrCM: broadcasting of cattle manure; BrPM: broadcasting of poultry manure; BaCM: subsurface banding of cattle manure; BaPM: subsurface banding of poultry manure; SCM: foliar spraying of cattle manure at two levels (1:5 and 1:10); SPM: foliar spraying of poultry manure at two levels (1:5 and 1:10). Means followed by the same letter are not significantly different according to LSD test (*p* < 0.05).

ANOVA indicated significant impacts of fertilizer treatments on the leaf area index (LAI) of buckwheat plants (*p* < 0.01; Table S2). The BaPM treatment resulted in the highest LAI (2), while the SCM(1:5) treatment yielded the lowest (0.44) (Figure 3a). Group comparisons of treatment means indicated notable differences in all comparisons (*p* < 0.01). Fertilized treatments enhanced the leaf area index (LAI) by 81% compared to the control, with UF exceeding organic fertilizers. Poultry manure treatments recorded a 126% higher LAI than their cattle manure counterparts. Subsurface banding methods improved the LAI by 47% over broadcasting methods, while subsurface banding and broadcasting applications achieved LAI values 92% higher than those from foliar spraying treatments (Table S2, Figure 3a).

Substantial differences for dry matter (DM) accumulation were found among fertilizer treatments (*p* < 0.01; Table S2). At 45 days after planting, UF and BaPM treatments produced the highest DM weights of 4869 and 4641 kg/ha, respectively (Figure 3b). Group comparisons at this growth stage showed that the UF treatment led to greater DM production compared to organic fertilizers. Additionally, subsurface banding and broadcasting applications enhanced DM by 30% relative to foliar spraying. Poultry manure treatments resulted in a 77% increase in DM over cattle manure treatments, while subsurface banding application techniques (BaCM, BaPM) caused a 42% increase compared to broadcasting (Table S2; Figure 3b).

**Figure 4:**
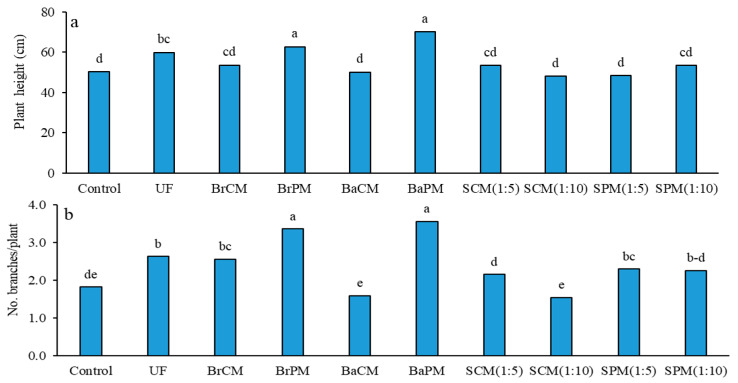
Effects of different fertilizer application methods on plant height (**a**) and number of branches per plant (**b**) in buckwheat. Control: without fertilizer; UF: urea fertilizer; BrCM: broadcasting of cattle manure; BrPM: broadcasting of poultry manure; BaCM: subsurface banding of cattle manure; BaPM: subsurface banding of poultry manure; SCM: foliar spraying of cattle manure at two levels (1:5 and 1:10); SPM: foliar spraying of poultry manure at two levels (1:5 and 1:10). Means followed by the same letter are not significantly different according to LSD test (*p* < 0.05).

Also shown were the significant effects of fertilizer treatments on plant height (*p* < 0.01; Table S2). The highest plant heights were achieved with BaPM (70 cm) and BrPM (62.7 cm) (Figure 4a). Group comparisons indicated that both subsurface banding and broadcasting applications produced plants that were 16% taller than those from foliar spraying treatments (SCM, SPM). Poultry manure treatments resulted in 14% taller plants compared to cattle manure treatments (Table S2; Figure 4a). No significant differences were noted in plant height between urea fertilizer and organic fertilizer treatments, nor between subsurface banding and broadcasting methods (Table S2; Figure 4a).

The significant effects of fertilizer treatments on the number of branches per plant were noteworthy (*p* < 0.01; Table S2). The highest branch counts were recorded in the BaPM (3.6) and BrPM (3.4) treatments (Figure 4b). Subsurface banding and broadcasting applications resulted in a 34% increase in branches compared to foliar spraying. Poultry manure treatments showed a 46% increase in branch number compared to cattle manure. No significant differences were identified between urea fertilizer and organic fertilizers, or between broadcasting and subsurface banding applications (Table S2; Figure 4b).

### 2.2. Seed Productivity

**Figure 5:**
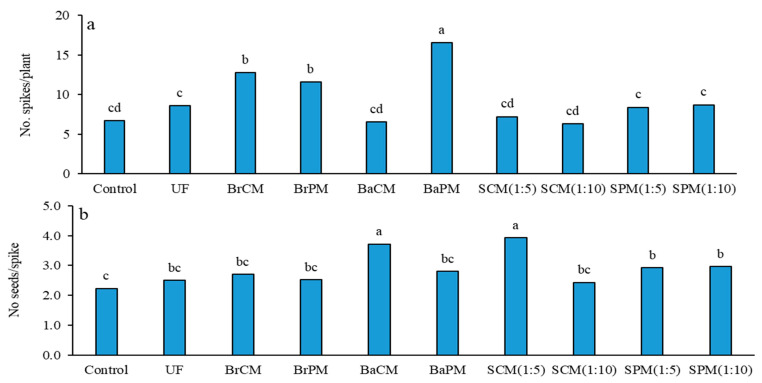
Effects of different fertilizer application methods on number of spikes per plant (**a**) and number of seeds per spike (**b**) in buckwheat. Control: without fertilizer; UF: urea fertilizer; BrCM: broadcasting of cattle manure; BrPM: broadcasting of poultry manure; BaCM: subsurface banding of cattle manure; BaPM: subsurface banding of poultry manure; SCM: foliar spraying of cattle manure at two levels (1:5 and 1:10); SPM: foliar spraying of poultry manure at two levels (1:5 and 1:10). Means followed by the same letter are not significantly different according to LSD test (*p* < 0.05).

Fertilizer treatments had a considerable impact on the number of spikes per plant (*p* < 0.01; Table S3). The greatest number of spikes per plant (17) was achieved with the BaPM treatment. In contrast, the lowest number of spikes per plant was recorded with the SCM(1:10) treatment, followed by the control and SCM(1:5) treatments (Figure 5a). Contrasts revealed that fertilization raised the number of spikes per plant by 43% in comparison to the control. Additionally, poultry manure treatments led to 38% more spikes per plant than cattle manure treatments, while subsurface banding and broadcasting applications increased spike counts by 55% compared to foliar spraying treatments (Table S3). No notable differences were detected between organic and inorganic fertilizer applications, nor between subsurface banding and broadcasting methods regarding the number of spikes per plant (Table S3; Figure 5a).

The quantity of seeds per spike was notably affected by fertilizer treatments (*p* < 0.01; Table S3). The highest seed count per spike was found with SCM(1:5) and BaCM treatments at 3.92 and 3.72, respectively (Figure 5b). The control treatment produced the lowest number of seeds per spike (2.23), which was statistically similar to the UF, BrPM, BrCM, BaPM, and SCM(1:10) treatments. Group comparisons showed significant increases in seed number per spike for fertilization versus control (+31%), organic compared to mineral fertilizers (+20%), cattle manure versus poultry manure (+14%), and subsurface application over broadcasting (+25%) (Table S3; Figure 5b).

**Figure 6:**
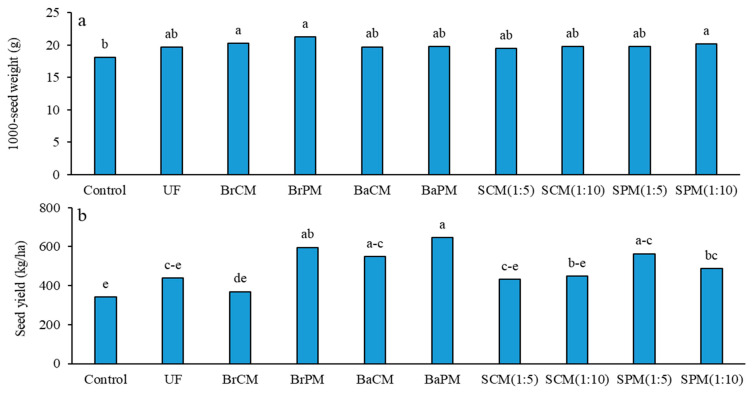
Effects of different fertilizer application methods on 1000-seed weight (**a**) and seed yield (**b**) in buckwheat. Control: without fertilizer; UF: urea fertilizer; BrCM: broadcasting of cattle manure; BrPM: broadcasting of poultry manure; BaCM: subsurface banding of cattle manure; BaPM: subsurface banding of poultry manure; SCM: foliar spraying of cattle manure at two levels (1:5 and 1:10); SPM: foliar spraying of poultry manure at two levels (1:5 and 1:10). Means followed by the same letter are not significantly different according to LSD test (*p* < 0.05).

No significant impact of fertilizer treatments on 1000-seed weight was found (Table S3). While the highest 1000-seed weight (21 g) was noted with broadcasting of poultry manure, it did not significantly differ from other fertilizer treatments, only showing a significant difference from the control (18 g) (Figure 6a). Group comparisons illustrated that fertilization enhanced the 1000-seed weight by 10% compared to the control, while other comparisons displayed no significant differences (Table S3; Figure 6a).

Significant effects of fertilizer treatments on buckwheat seed yield were indicated (*p* < 0.01; Table S2). The highest seed yield (646 kg/ha) was recorded with BaPM, which was not significantly different from the BrPM (596 kg/ha), BaCM (549 kg/ha), and SPM(1:5) (564 kg/ha) treatments. The control treatment resulted in the lowest seed yield (340 kg/ha) (Figure 6b). Group comparisons indicated significant increases in seed yield for fertilization versus control (48%), poultry manure over cattle manure treatment (27%), and subsurface application compared to broadcasting (24%). No significant differences were found between organic and mineral fertilizers or between subsurface banding and broadcasting applications and foliar spraying (Table S3; Figure 6b).

### 2.3. Seed Nutrient Value

**Figure 7:**
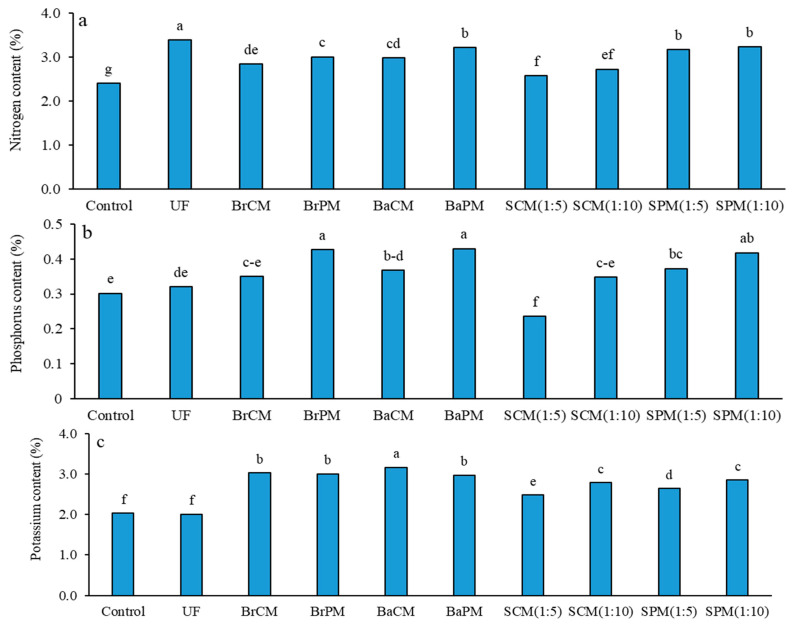
Effects of different fertilizer application methods on contents of nitrogen (**a**), phosphorus (**b**), and potassium (**c**) in buckwheat seeds. Control: without fertilizer; UF: urea fertilizer; BrCM: broadcasting of cattle manure; BrPM: broadcasting of poultry manure; BaCM: subsurface banding of cattle manure; BaPM: subsurface banding of poultry manure; SCM: foliar spraying of cattle manure at two levels (1:5 and 1:10); SPM: foliar spraying of poultry manure at two levels (1:5 and 1:10). Means followed by the same letter are not significantly different according to LSD test (*p* < 0.05).

Seed nitrogen content was significantly affected by various fertilizer treatments (Table S4). Treatment mean comparisons indicated that the highest seed nitrogen content (3.4% in DM) was seen in the UF treatment, while the lowest (2.4%) was recorded in the control treatment (Figure 7a). Subsurface banding and broadcasting applications increased seed nitrogen by 3% compared to foliar spraying treatments. Poultry manure treatments raised seed nitrogen by 13% over cattle manure treatments (Figure 7a). Urea fertilizer increased seed nitrogen by 13% compared to organic fertilizers. Furthermore, subsurface application raised seed nitrogen by 7% compared to broadcasting methods (Table S4; Figure 7a).

Notable variations in seed phosphorus content were shown across the experimental treatments at the 1% significance level (Table S4). The highest phosphorus level (0.43% in DM) was linked to the use of poultry manure, in both BaPM and BrPM treatments. Conversely, the lowest phosphorus level (0.23%) was noted with SCM(1:5) (Figure 7b). Fertilization enhanced seed phosphorus content by 20% compared to the control. Subsurface banding and broadcasting applications raised phosphorus content by 14% relative to foliar spraying treatments (SCM, SPM). Poultry manure applications led to a 27% increase in phosphorus compared to cattle manure (Table S4; Figure 7b).

The results in Table S4 indicate significant differences in seed potassium content among different fertilizer treatments. The highest potassium content (3%) was achieved with BaCM, whereas the lowest level (2%) was observed in both the UF and control treatments (Figure 7c).

Subsurface banding and broadcasting treatments increased potassium levels by 13% when compared to foliar spraying treatments. Organic fertilizers raised the potassium content by 43% in comparison to UF treatment. Yet no significant differences were detected between poultry manure and cattle manure applications or between subsurface banding and broadcasting methods (Table S4; Figure 7c).

**Figure 8:**
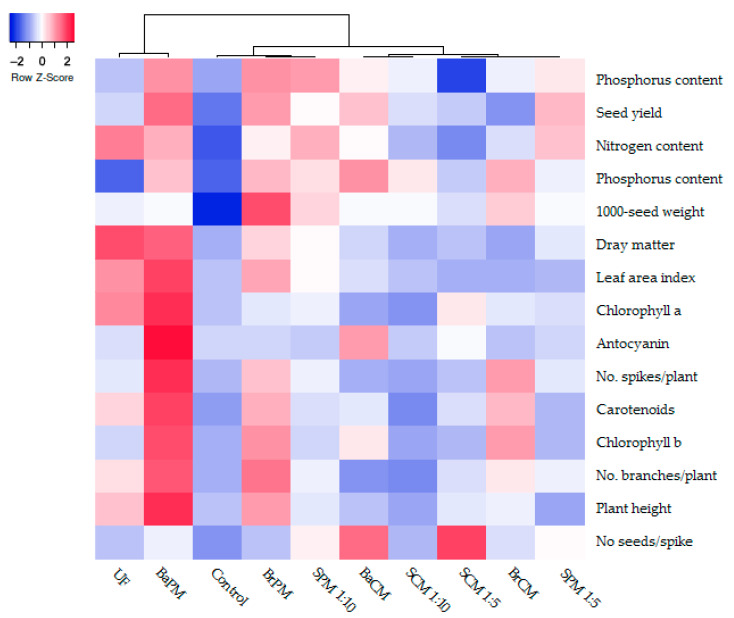
Hierarchical clustering dendrogram and heat map for photosynthetic pigments, anthocyanin levels, growth, yield, and nutritional quality in buckwheat through optimized application methods for organic fertilizers under different levels of animal manures that were significantly different (*p* < 0.05 after LSD post-hoc testing). Colors represent a relative scale (−2 to +2). Darker blue indicates lower values, while darker red indicates higher values. Control: without fertilizer; UF: urea fertilizer; BrCM: broadcasting of cattle manure; BrPM: broadcasting of poultry manure; BaCM: subsurface banding of cattle manure; BaPM: subsurface banding of poultry manure; SCM: foliar spraying of cattle manure at two levels (1:5 and 1:10); SPM: foliar spraying of poultry manure at two levels (1:5 and 1:10). Means followed by the same letter are not significantly different according to LSD test (*p* < 0.05).

The results of the hierarchical clustering dendrogram and heat map (Figure 8) indicate that the BaPM and UF treatments are categorized within the same cluster. Similarly, the BrPM, SPM(1:10), and control treatments are also grouped together. Other fertilizer treatments form an additional cluster. Notably, the BaPM, BrPM, and UF treatments exhibit a positive and significant correlation in several parameters, including the number of branches per plant, plant height, leaf area index, photosynthetic pigments, dry matter, and nitrogen content. With the exception of seed weight and number of seeds per spike, BaPM demonstrates superior values across most measured traits (Figure 8).

## 3. Discussion

### 3.1. Effect of Fertilizer Application Methods on Photosynthetic Pigments and Anthocyanin

Subsurface banding of poultry manure showed a superior performance across all pigment parameters, highlighting the significance of both the fertilizer type and application method. The increase in chlorophyll content, especially with organic fertilizers (Figure 1), is likely due to improved nitrogen availability in the rhizosphere. Fertilizer application affects assimilation pigments in *Festuca* spp. turf leaves [23], with nitrogen playing a key role in chlorophyll synthesis. Both organic and inorganic fertilizers enhance chlorophyll content, but poultry manure outperforms cattle manure due to the higher N concentration and faster mineralization, providing more readily available nitrogen for plant uptake [24]. Subsurface banding increases chlorophyll content more effectively than broadcasting due to localized nutrient placement, reducing loss and enhancing root–nutrient interactions [13]. This method improves chlorophyll and nitrogen contents in maize (*Zea mays*) plants by conserving litter-derived nitrogen [25], creating a favorable rhizosphere environment for nutrient uptake and chlorophyll synthesis. Increased carotenoid content suggests improved light utilization and stress tolerance [26]. The notable rise in anthocyanins, especially with subsurface banding of poultry manure (Figure 2b), indicates enhanced plant stress tolerance and potential improvements in crop nutritional properties [27]. These increases suggest that organic fertilizers (like poultry manure) and subsurface banding may boost buckwheat’s stress resilience and seed mineral content.

Subsurface banding and broadcasting applications outperformed foliar spraying in enhancing pigment content, indicating more effective nutrient uptake through roots. This continuous nutrient supply via root absorption contrasts with intermittent foliar applications, generally proving more effective for plant metabolism and pigment synthesis [28]. In our case, the total amounts of nutrients sprayed on the canopies were only 50% of those applied with manure, which likely contributed to the observed differences in effectiveness. Repeated foliar applications could potentially bridge this gap; however, it is essential to take into account the extra expense associated with each foliar treatment.

### 3.2. Effect of Fertilizer Application Methods on Growth Parameters

Fertilizer treatments significantly impacted the buckwheat’s growth indicators, highlighting the importance of effective nutrient management. Subsurface banding of poultry manure consistently outperformed other treatments, emphasizing the importance of both the fertilizer type and application method in enhancing plant growth and development. Organic fertilization, particularly subsurface banding of poultry manure, significantly increases the leaf area index due to improved nitrogen availability and uptake, enhancing leaf expansion and canopy development in various crops. This boost in leaf area index improves light interception and photosynthetic capacity, supporting greater biomass production [29]. In maize, poultry manure application increases the leaf number and leaf area index, correlating positively with grain yield [30]. Chicken-manure-processed organic fertilizer exhibits an accelerated rate of nitrogen mineralization, rendering it ideal for crops with brief growth cycles. Additionally, its elevated nitrogen concentration and capacity to raise soil pH improve nutrient accessibility and promote plant development [31].

Leaf surface area, which captures solar radiation, along with chlorophyll *a*, is integral for transforming solar energy into photosynthetic compounds and consequently facilitating the buildup of dry matter. In the present study, it was noted that the application of urea fertilizer and the subsurface application of poultry manure enhanced leaf area and promoted chlorophyll production, thereby contributing positively to the accumulation of dry matter. Poultry manure increased dry matter weight in *Andrographis paniculata* [29], moringa (*Moringa oleifera* Lam.) [32], and sweet basil (*Ocimum basilicum* L.) [33]. In buckwheat, broiler litter combined with mineral fertilizers increased above-ground dry matter by 56% compared to mineral fertilizers alone [22]. Subsurface banding of poultry manure improved forage yield by 40% in perennial bermudagrass (*Cynodon dactylon* L. Pers) while reducing nutrient runoff by 90% [34], demonstrating its efficacy in enhancing biomass production and environmental sustainability across diverse crop species.

In this study, the positive correlation between leaf area index (LAI) and dry matter (DM) accumulation highlights their interconnected nature. Increased LAI, especially with organic fertilization and subsurface banding of poultry manure, corresponded with higher DM. This relationship reflects the enhanced photosynthetic capacity from greater leaf area, driving increased biomass production and overall plant productivity.

Poultry manure positively impacted plant height in our study, aligning with previous research on various crops. Animal manure enhances growth-regulating hormones, nutrient retention, and beneficial microorganism activity [35]. Both surface and subsurface applications of cattle manure maximized plant height in *Allium sativum* [15], while poultry manure and integrated fertilizers significantly affected *Nigella sativa* height [36]. Fertilizer type and application method influenced branch development, with subsurface banding or broadcasting of poultry manure being most effective, likely due to increased nitrogen availability.

### 3.3. Effects of Fertilizer Application Methods on Yield Components and Seed Yield

The enhancement of the number of spikes in buckwheat through subsurface banding of poultry manure aligns with prior studies on the efficiency of organic fertilizers in promoting spike development. For instance, Salehi et al. [6] noted an increase in spike count in buckwheat that was treated with broiler litter when compared to buckwheat receiving mineral fertilizers. Increased spike creation likely stems from the higher nitrogen content in poultry manure, crucial for plant reproductive growth. Foliar spraying of cattle manure (1:5) and subsurface banding of cattle manure increased seeds per spike, possibly due to reduced physiological sinks from fewer spikes per plant (Figure 5), allowing greater resource allocation to seed setting. This aligns with Khatate et al. [37], who reported that foliar application of cattle urine positively impacts wheat growth, increasing grains per spike. Organic fertilizers’ beneficial effects on fruit development extend to various crops, as Basay et al. [38] found that organic manures enhance success in organic seed production in *Solanum melongena* (L.) due to improved plant nutrition.

Broadcasting of poultry manure resulted in the highest 1000-seed weight (21 g) in buckwheat, possibly due to prolonged green stay, but this only differed significantly from the control (Figure 6a). This aligns with findings in *Vigna unguiculata* (L.), where different levels of farmyard manure did not significantly differ from chemical fertilizers [39], while broiler litter application increased the 1000-seed weight in buckwheat compared to mineral fertilizer [6]. These results suggest that fertilizer treatments may influence 1000-seed weight, but effects often lack statistical significance across fertilizer types and application methods. This study reveals that fertilizer treatments significantly influenced buckwheat seed yield, with poultry manure outperforming mineral fertilizers and the control (Figure 7b). Subsurface banding of poultry manure yielded 646 kg/ha, attributed to its nutrient-rich composition and immediate nitrogen availability, enhancing photosynthetic capacity (Figure 1 and Figure 2). Hsu and Lai [31] support these findings, reporting that organic fertilizers improve soil quality and nutrient availability. Subsurface banding techniques reduce nutrient losses by up to 90% and improve crop yields by 40–80% [34,40]. Broadcasting poultry manure and subsurface banding of cattle manure also demonstrated effectiveness, attributed to enhanced soil health through increased beneficial microorganism activity, improved nutrient availability, and increased water holding capacity [41]. Fertilization increased seed yield by 48% over the control, with poultry manure outperforming cattle manure by 27%, likely due to its lower C/N ratio (Table 1) and higher N content, allowing for faster decomposition and nutrient release [42]. These results align with Salehi et al. [22], who reported increased yields of buckwheat and fenugreek (*Trigonella foenum-graecum*) with broiler litter application compared to mineral fertilizers. Subsurface banding outperformed broadcasting with a 24% yield increase, aligning with findings by Amin et al. [15] in garlic and similar results in *Cynodon dactylon* (L.) [34] and *Zea mays* (L.) [12]. This method enhances nutrient retention and availability in the root zone, improving plant uptake while reducing losses from runoff and volatilization. Concentrated nutrient placement increases efficiency, potentially allowing for lower application rates compared to broadcasting [12,34]. The comparable performance of organic and mineral fertilizers suggests that well-managed organic fertilization can match or exceed mineral fertilizers, offering significant advantages for sustainable agriculture.

### 3.4. Effect of Fertilizer Application Methods on Seed Macronutrient Content

Our findings highlight the critical role of fertilizer type and application method in enhancing the macronutrient content of buckwheat seeds, particularly nitrogen, phosphorus, and potassium (Figure 7). Urea fertilizer resulted in a higher nitrogen content due to its rapid availability [43], consistent with Salehi et al. [22], who reported the highest nitrogen concentration in buckwheat with urea compared to organic fertilizers like broiler litter. In contrast, poultry manure (Figure 7) provides a nitrogen mineralization rate ranging from 15% to 55% across different temperatures, which exceeds that observed in organic fertilizers [44]. Subsurface banding facilitates gradual nutrient release from decomposing manure while minimizing leaching losses [45]. This characteristic results in a more effective uptake and distribution of elevated nitrogen levels to the vegetative parts and seeds. However, the use of urea can lead to an imbalance between vegetative and reproductive growth, resulting in increased grain nitrogen concentration [46]. Additionally, the slower decomposition rates associated with cattle manure may limit its effectiveness in providing readily available nitrogen compared to poultry manure. This highlights the importance of considering the C/N ratio in organic fertilizers, as it directly impacts microbial activity and nitrogen mineralization. Overall, poultry manure may offer more readily available nutrients, enhancing its effectiveness in nutrient management.

Phosphorus is essential for energy transfer and metabolic processes in plants. The higher phosphorus content associated with poultry manure applications (Figure 7b) indicates that this organic source enhances phosphorus availability more effectively than cattle manure or mineral fertilizers. Similarly, Salehi et al. [22] reported higher phosphorus content and uptake in buckwheat and fenugreek seeds following broiler litter application. Singh et al. [47] found a significant increase in phosphorus concentration in basil with an integrated fertilizer system compared to mineral fertilizers, with the highest concentration (0.7%) observed in that system. Using animal manure for buckwheat nutrition adds phosphorus and other nutrients (Table 1), increases organic matter, enhances soil cation exchange capacity, and prevents phosphorus fixation. Consequently, sufficient nitrogen and phosphorus become available, enhancing nutrient uptake and seed quality [48]. The higher phosphorus content in buckwheat seeds after applying poultry manure at a 1:10 ratio (Figure 7b) suggests that foliar application of poultry manure extract is particularly beneficial in phosphorus-deficient conditions [9]. Foliar phosphorus application can significantly increase biomass, phosphorus concentration, and yield in wheat and maize under deficient conditions [49], with effects more pronounced in low-phosphorus soils. This finding highlights the relevance of organic amendments like poultry manure in sustainable agriculture, as they can improve phosphorus availability and reduce reliance on mineral fertilizers, thereby mitigating environmental impacts associated with phosphorus runoff.

Potassium is vital for regulating plant physiological processes, such as water retention and stress tolerance [50]. Our results show that both poultry and cattle manures effectively supply potassium through subsurface banding or broadcasting, with no significant differences between the two methods, providing farmers with flexibility without compromising potassium availability. However, subsurface banding of cattle manure enhances nutrient retention, as demonstrated by Hulugalle et al. [51], who found that applying cattle manure at 16 t/ha improved potassium availability by 0.16 t/ha and reduced the exchangeable sodium percentage in soils. In terms of grain potassium content, a different trend was noted compared to phosphorus (Figure 7b,c). The high potassium volume in cattle manure (Table 1), particularly under band placement conditions that minimize fertilizer loss, likely contributed to increased uptake and accumulation in the grain. While foliar application of manure extract improved the grain potassium content, it was lower than that from poultry manure application and surface application of cattle manure. Singh et al. [47] similarly reported the highest potassium uptake in basil with an integrated system of cattle manure and mineral fertilizer.

## 4. Materials and Methods

### 4.1. Site Description and Climatic Properties

**Figure 9:**
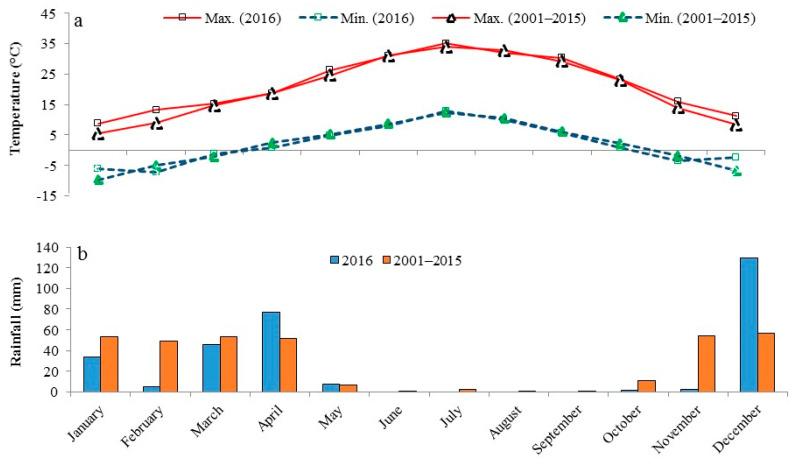
Climatic conditions during the experimental period (2016) compared to the 15-year average (2001–2015): (**a**) minimum and maximum air temperatures and (**b**) monthly rainfall.

The filed study was conducted at the Research Farm of the Faculty of Agriculture, Shahrekord University. The research site is situated at an elevation of 1148 m above sea level, at coordinates 33°44′ N and 48°28′ E. According to the Köppen classification, the region has a Mediterranean climate, characterized by moderate temperatures, rainy conditions, and hot, dry summers. Figure 9 illustrates the monthly rainfall and minimum and maximum air temperatures during the experimental period.

The soil at the research site is classified as silty clay loam. Prior to seed bed preparation in the spring, a composite soil sample was collected from a depth of 0 to 30 cm and analyzed for its chemical and physical properties. Additionally, the study investigated the chemical characteristics of the animal manures used in the experiment. Poultry and cattle manures were sourced from the university’s poultry and cattle farms before the experiment commenced. These manure samples were then sent to the Shahrekord Research Center laboratory for chemical analysis. Table 1 provides comprehensive details on the chemical properties of the soil at the research site, along with the characteristics of the cattle and poultry manures, as well as their respective extracts used in the study.

Standard laboratory procedures were used to determine the chemical properties of the soil, manure, and extracts. Soil pH was measured in a 1:2.5 soil/water suspension using a calibrated pH meter. Electrical conductivity (EC) was determined in a saturated paste extract using a conductivity meter [52]. Organic carbon (OC) in soil and manure was determined by the Walkley–Black wet oxidation method [53]. Total nitrogen (N) was measured using the Kjeldahl method [54]. Available phosphorus (P) was extracted via the sodium bicarbonate (Olsen) method and determined colorimetrically using a spectrophotometer [55]. Exchangeable potassium (K) was extracted with 1.0 N ammonium acetate (pH 7.0) and quantified using flame photometry [56]. Available micronutrients were extracted using the DTPA method [57] and measured by atomic absorption spectroscopy (AAS). Parameters in manure extracts were analyzed using relevant standard methods for solutions.

### 4.2. Field Operations and Treatments

The field experiment was conducted using a randomized complete block design (RCBD) with three replications. In mid-May 2016, the process of seed bed preparation commenced. The initial step involved plowing the land, followed by two rounds of disking. Subsequently, plots measuring 3 × 2 m were established, each containing 10 planting rows with 25 cm spacing between them. To prevent water mixing between plots, a one-meter gap was maintained between blocks, and each block was equipped with its own drainage channel. The experimental layout consisted of 30 plots in total, representing 10 treatments replicated across three blocks.

Following seed bed preparation, buckwheat seeds were sown on 12 May at a depth of 3 cm and immediately irrigated. Further irrigation was conducted based on environmental conditions and plant requirements. When plants reached the 4-leaf stage, thinning was performed to achieve a target density of 240 plants per square meter.

The experimental treatments encompassed various fertilization methods. These included two approaches for applying cattle manure (broadcasting and subsurface banding; BrCM and BaCM), two methods for applying poultry manure (broadcasting and subsurface banding; BrPM and BaPM), two concentrations of foliar spraying using cattle manure extract (SCM(1:5) and SCM(1:10)), two levels of foliar spraying using poultry manure extract (SPM(1:5) and SPM(1:10)), urea fertilizer (UF), and a control treatment using water foliar spray. A schematic representation of the experimental layout showing the distribution of these treatments within each block of the RCBD is presented in Figure S1. The quantity of poultry and cattle manures was calculated to provide 60 kg N/ha, taking into account the nitrogen mineralization efficiency (50% for total N in solid manure and 100% for extracts). Cattle and poultry manure extracts were prepared by mixing fresh manure with distilled water at ratios of 1:5 and 1:10 (*w*/*v*). The mixtures were stirred thoroughly, left to stand at room temperature for three days, and filtered through a cheesecloth to obtain foliar spray solutions. Cattle and poultry manures were selected due to their prevalence in local agricultural practices and their distinct nutrient profiles relevant to optimizing organic fertilization. Urea fertilizer was applied at a rate of 130 kg/ha concurrently with the sowing of seeds, with urea’s 46% nitrogen content resulting in a pure nitrogen application rate of approximately 60 kg N/ha in the UF treatment. For both band and broadcast applications, as well as urea treatments, fertilizers were applied to each plot one day prior to planting. In the band application method, a furrow was created to a depth of 10 cm, into which the fertilizers were placed before being covered with soil. In contrast, for the broadcast application, the fertilizers were integrated into the soil through raking. Foliar spray treatments were administered on 12 June (late vegetative/early flowering stage) and 27 June (peak flowering). During each application, plants were sprayed with the respective solution until the foliage was thoroughly wetted, reaching the point of incipient runoff. This method ensured consistent coverage appropriate to plant size across these treatments. Throughout the growth period, necessary maintenance tasks such as weeding and breaking soil crusts were carried out homogeneously across all plots.

### 4.3. Measurements

#### 4.3.1. Chlorophyll Determination

Leaf chlorophyll concentration was assessed using the method described by Porra [58] during the full flowering stage. Fresh leaf tissue (1 g) was weighed, finely chopped, and thoroughly homogenized in a porcelain mortar with 80% acetone. The homogenate was brought to a final volume of 25 mL with 80% acetone. The resulting solution was centrifuged at 5000 rpm for 15 min. Spectrophotometric measurements of the leaf extract were taken at 663.6 and 646.6 nm. Chlorophyll *a* and *b* concentrations were calculated using the following equations [58]:

(1) $$\mathrm{Chl}\;a\;(\mathrm{µg}/\mathrm{mL})=12.25(A_{663.6})-2.55(A_{646.6})$$

(2) $$\mathrm{Chl}\;b\;(µg/mL)=20.31(A_{646.6})-4.91(A_{663.6})$$

where Chl *a*, Chl *b*, A_663.6_, and A_646.6_ represent chlorophyll *a*, chlorophyll *b*, and the absorbance at 663.6 and 646.6 nm, respectively.

#### 4.3.2. Carotenoid and Anthocyanin Estimations

Carotenoid and anthocyanin concentrations were determined using the method of Sims and Gamon [59]. The following equations were employed:

(3) $$\mathrm{Anthocyanin}\;(\mathrm{µmol}/\mathrm{mL})=0.08173\;A_{537}\;-\;0.00697\;A_{647}\;-\;0.002228\;A_{663}$$

(4) $$Carotenoids\;(µmol/mL)=[(A_{470}-\left(17.1\times \left(Chl\;a+Chl\;b\right)-9.479\times anthocyanin\right)]/119.26$$

where A_537_, A_647_, A_663_, and A_470_ represent the absorbance at 537, 647, 663, and 470 nm, respectively.

#### 4.3.3. Leaf Area Index Measurement

Leaf area index (LAI) is a critical parameter for characterizing crop canopy in relation to the cultivated area. In this study, the LAI measurement procedure was conducted as follows: at 45 days after planting, five buckwheat plants were randomly selected for analysis. The leaf area of these plants was measured using a leaf area meter (Model AM 200, manufactured by ADC BioScientific Ltd., Hoddesdon, UK). Following the method described by Jiang et al. [60], the LAI was measured 45 days after planting. The LAI was calculated using the following equation:

(5) $$\mathrm{LAI}\;=\;\frac{LA}{GA}$$

where LAI represents the leaf area index, LA is the leaf area, and GA denotes the ground area.

#### 4.3.4. Dry Matter (DM)

Forty-five days after planting, five buckwheat plants were randomly selected from the experimental plots. The samples (leaves plus stems) were oven-dried at 72 °C for 48 h and subsequently weighed using a precision balance, following the method described by Fallah et al. [61].

#### 4.3.5. Plant Height and Branches Number

At the physiological maturity stage on 2 August, ten plants were randomly selected from each plot. The average height (cm) of the plants and the number of branches per plant were subsequently measured.

#### 4.3.6. Yield and Yield Components

To assess yield components, ten plants were randomly selected at physiological maturity from each plot. These samples were used to determine the number of spikes per plant, seeds per spike, and 1000-seed weight. For the evaluation of seed yield per unit area, border effects were mitigated by excluding the two outermost rows and 25 cm from both ends of each plot. The remaining plants, encompassing an area of 2 square meters, were harvested. Grain yield was subsequently calculated based on dry weight and expressed in kilograms per hectare (kg/ha).

#### 4.3.7. Seed Macronutrient Concentration

The measurement of macronutrients (nitrogen, phosphorus, and potassium) in buckwheat seeds was conducted as follows: after harvesting, 20 g of seeds were randomly selected from the grain yield. These samples were dried in an oven at 70 °C for 48 h. The dried samples were then ground and sieved through a 2-millimeter mesh. Subsequently, total nitrogen was determined using the Kjeldahl method [54]. Phosphorus was measured using a spectrophotometer (Novaspec-LKB Pharmacia-11 model) following the Olsen and Sommers method [55]. Potassium was quantified using a flame photometer (Jenway-Pfp7 model) as described by Simard [56]. These procedures allowed for the precise determination of macronutrient concentrations in the buckwheat grain samples, providing valuable data on mineral content and agronomic performance of the crop under the studied conditions.

### 4.4. Statistical Analysis

Statistical analysis of the data for the evaluated parameters in this experiment was performed using the SAS V9 software in a randomized complete block design. Treatment means were compared using the LSD test at a 5% probability level, and we tested orthogonal contrasts (independent linear comparisons) for group means to examine specific patterns of differences. Data visualizations were conducted using Microsoft Word and Excel. The hierarchical clustering dendrogram and heat map for plant growth, yield, and nutrient contents of buckwheat under different animal manure treatments were generated using Heatmapper [62]. All treatments showed significant differences in percentage (*p* < 0.05) after LSD post-hoc testing.

## 5. Conclusions

Subsurface banding of poultry manure significantly enhanced buckwheat’s photosynthetic pigments, growth, yield, and seed macronutrient content, outperforming broadcast application and synthetic urea. Banded cattle manure notably increased seed potassium (3%). Foliar application of poultry manure extract (1:5) effectively addressed phosphorus limitations. These findings demonstrate that optimizing organic fertilizer application methods—such as the strategic subsurface banding of poultry manure used in this study—can enhance nutrient retention, crop yields, and sustainable agricultural practices. Our results show that banded poultry manure synergistically improves yield, nutrient efficiency, and ecological resilience, outperforming non-optimized approaches like one-off basal fertilization, which may increase biomass without improving economic yield. Future research should explore molecular mechanisms and field-scale validation under diverse agroclimatic conditions.

## Figures and Tables

**Table 1:** Selected chemical properties of soil and manure used in the experiment.

Parameter	Unit	Soil	Poultry Manure (PM)	Cattle Manure (CM)	PM Extract (1:5)	PM Extract (1:10)	CM Extract (1:5)	CM Extract (1:10)
EC	µS/cm	4000	8650	11,360	11,170	6740	14,060	12,300
pH	-	7.81	6.76	5.86	8.45	8.46	5.05	6.24
Nitrogen	g/kg	0.80	33.9	3.9	25.6	12.8	12.8	9.8
Phosphorus	mg/kg	15.3	16,100	9800	11,400	5700	5200	2600
Potassium	mg/kg	120	15,800	34,900	10,300	5100	16,400	8200
Sodium	mg/kg	-	1.99	1.98	2.36	1.18	2.97	1.48
OC	g/kg	7.0	69.5	17.5	-	-	-	-
Iron	mg/kg	0.03	976	324	922	461	812	406
Zinc	mg/kg	0.05	80.4	80.4	321	160	115	57.0
Copper	mg/kg	0.08	86.0	19.0	63.0	31.5	30.0	14.5
Manganese	mg/kg	0.04	411	109	215	107	102	51.2

## Data Availability

The datasets generated during the current study are available from the corresponding author on reasonable request.

## Supplementary Materials

The following supporting information can be downloaded at: https://www.mdpi.com/article/10.3390/plants14172700/s1, Table S1: Statistical analysis for manure application method effects on chlorophyll, carotenoid, and anthocyanin in buckwheat; Table S2: Statistical analysis for manure application method effects on leaf area index, dry matter accumulation, plant height, and number of branches per plant in buckwheat; Table S3: Statistical analysis for manure application method effects on seed yield and yield components in buckwheat; Table S4: Statistical analysis for manure application method effects on macronutrients content in buckwheat. Figure S1: Experimental layout of the randomized complete block design (RCBD) with three blocks, each containing all ten treatments. Control: without fertilizer; UF: urea fertilizer; BrCM: broadcasting of cattle manure; BrPM: broadcasting of poultry manure; BaCM: subsurface banding of cattle manure; BaPM: subsurface banding of poultry manure; SCM: foliar spraying of cattle manure at two levels (1:5 and 1:10); SPM: foliar spraying of poultry manure at two levels (1:5 and 1:10).
