# Supplementary material for: Subsurface Banding of Poultry Manure Enhances Photosynthetic Efficiency, Yield, and Nutrient Uptake in Buckwheat
Source: Plants (Basel). 2025 Aug 29;14(17):2700. doi: 10.3390/plants14172700 (PMC12430484; doi:10.3390/plants14172700)
Supplement: Supplementary file 1 [file plants-14-02700-s001.zip › plants-3792638-supplementary.pdf]

Table S1. Statistical analysis for manure application method effects on chlorophyll, carotenoid and anthocyanin in buckwheat.

Source of variation	df	Chlorophyll *a*	Chlorophyll *b*	Carotenoids	Anthocyanin
Replication	2	NS§	NS	NS	NS
Treatment	9	**	**	**	**
EMS¶	18	0.75	0.16	0.02	1×10^{-6}
CV (%)		16.5	7.9	10.5	10.2
			<u>Contrast</u>		
Fertilizer vs. control		(+53%)**	(+337%)**	(+109%)**	(+127%)**
Organic vs. inorganic		(-39%)**	(+85%)**	(-14%)NS	(+78%)**
Solid vs. extract		(+30%)**	(+387%)**	(+110%)**	(+205%)**
Band vs. broadcast		(+44%)**	(-1.3%)NS	(+2.5%)NS	(+695%)**
Poultry vs. cattle		(+55%)**	(+48%)**	(+41%)**	(+45%)**

** Significant at the $p < 0.01$ probability level.
§ NS, not significant at the $p < 0.05$ probability level.
¶ EMS = error mean square.
The numbers within the parentheses represent the percentage increase (+) and decrease (-) of the initial group in relation to the subsequent group.

Table S2. Statistical analysis for manure application method effects on leaf area index, dry matter accumulation, plant height, and number of branches per plant in buckwheat.

Source of variation	df	Leaf area index	Dry matter accumulation	Plant height	No. branches/plant
Replication	2	NS§	NS	NS	NS
Treatment	9	**	**	**	**
EMS¶	18	0.03	25.3	26.9	0.06
CV (%)		17.5	18.5	9.4	10.6
			<u>Contrast</u>		
Fertilizer vs. control		(+81%)*	(+61%)**	(+10%)NS	(+33%)**
Organic vs. inorganic		(-44%)**	(-48%)**	(-8%)NS	(-8%)NS
Solid vs. extract		(+92%)**	(+30%)**	(+16%)**	(+34%)**
Band vs. broadcast		(+47%)**	(+42%)**	(+3%)NS	(-13%)NS
Poultry vs. cattle		(+126%)**	(+77%)**	(+14%)**	(+46%)**

* Significant at the $p < 0.05$ probability level.
** Significant at the $p < 0.01$ probability level.
§ NS, not significant at the $p < 0.05$ probability level.
¶ EMS = error mean square.
The numbers within the parentheses represent the percentage increase (+) and decrease (-) of the initial group in relation to the subsequent group.

Table S3. Statistical analysis for manure application method effects on seed yield and yield components in buckwheat.

Source of variation	df	No. spikes/plant	No. seeds/spike	1000-seed weight	Seed yield
Replication	2	NS§	NS	NS	NS
Treatment	9	**	**	NS	**
EMS¶	18	2.0	0.13	1.11	5307
CV (%)		15.1	12.7	5.3	15.1
			Contrast		
Fertilizer vs. control		(+44%)**	(+32%)**	(+11%)**	(+50%)**
Organic vs. inorganic		(+13%)NS	(+19%)**	(+2%)NS	(+17%)NS
Solid vs. extract		(+55%)**	(-4%)NS	(+2%)NS	(+12%)NS
Band vs. broadcast		(-5%)NS	(+25%)**	(-5%)NS	(+24%)*
Poultry vs. cattle		(+38%)**	(-12%)**	(+2%)NS	(+28%)**

* Significant at the $p < 0.05$ probability level.
** Significant at the $p < 0.01$ probability level.
§ NS, not significant at the $p < 0.05$ probability level.
¶ EMS = error mean square.
The numbers within the parentheses represent the percentage increase (+) and decrease (-) of the initial group in relation to the subsequent group.

Table S4. Statistical analysis for manure application method effects on macronutrients content in buckwheat.

Source of variation	df	Nitrogen content	Phosphorous content	Potassium content
Replication	2	NS§	NS	NS
Treatment	9	**	**	**
EMS¶	18	78×10^{-4}	9×10^{-4}	38×10^{-4}
CV (%)		2.9	8.5	2.3
			Contrast	
Fertilizer vs. control		(+25%)**	(+20%)**	(+37%)**
Organic vs. inorganic		(-12%)**	(+15%)**	(+42%)**
Solid vs. extract		(+3%)*	(+14%)**	(+13%)**
Band vs. broadcast		(+6%)**	(+3%)NS	(+2%)NS
Poultry vs. cattle		(+13%)**	(+27%)**	(+0.1%)NS

* Significant at the $p < 0.05$ probability level.
** Significant at the $p < 0.01$ probability level.
§ NS, not significant at the $p < 0.05$ probability level.
¶ EMS = error mean square.
The numbers within the parentheses represent the percentage increase (+) and decrease (-) of the initial group in relation to the subsequent group.

Block 1	BaPM	SPM (1:10)	BrCM	SPM (1:5)	Control	UF	SCM (1:5)	BaCM	BrPM	SCM (1:10)
Block 2	BrCM	SPM (1:10)	Control	BrPM	BaCM	SCM (1:5)	UF	SPM (1:5)	BaPM	SCM (1:10)
Block 3	SPM (1:5)	BaPM	SCM (1:10)	Control	SCM (1:5)	UF	BrPM	BrCM	SPM (1:10)	BaCM

Figure. S1. Experimental layout of the randomized complete block design (RCBD) with three blocks, each containing all ten treatments. Control: without fertilizer; UF: urea fertilizer; BrCM: broadcasting of cattle manure; BrPM: broadcasting of poultry manure; BaCM: subsurface banding of cattle manure; BaPM: subsurface banding of poultry manure; SCM: foliar spraying of cattle manure at two levels (1:5 and 1:10); SPM: foliar spraying of poultry manure at two levels (1:5 and 1:10).
